# Palmitic Acid Induces MicroRNA-221 Expression to Decrease Glucose Uptake in HepG2 Cells via the PI3K/AKT/GLUT4 Pathway

**DOI:** 10.1155/2019/8171989

**Published:** 2019-11-11

**Authors:** Fang Huang, Jie Chen, Jingwen Wang, Pingping Zhu, Wenting Lin

**Affiliations:** School of Public Health, Fujian Medical University, Xueyuan Road 1, Minghou District, Fuzhou City, Fujian Province, China

## Abstract

Obesity-related insulin resistance and high fatty acid concentrations occur during the development of type 2 diabetes mellitus. The role of high concentrations of plasma-free fatty acids is not fully understood. In this study, palmitic acid (PA, 0.8 mM for 24 h) induced the expression of miR-221 that bound to phosphoinositide 3-kinases (PI3K) mRNA to inhibit glucose uptake by HepG2 cells. Compared with controls, PA significantly decreased glucose uptake, increased insulin receptor substrate-2 (IRS-2) and miR-221 expression, and decreased phosphoinositide 3-kinase (PI3K), protein kinase B (AKT), and glucose transporter type 4 (GLUT4) mRNA expression. Luciferase reporter assay revealed that miR-221 binding inhibited PI3K expression. Transfection of HepG2 cells with an miR-221 mimic induced miR-221 expression and inhibited the PI3K/AKT pathway. PA decreased glucose uptake in HepG2 cells by inducing the expression of miR-221, which bound to PI3K mRNA and suppressed PI3K/AKT signaling. miR-221 may be a novel target for preventing and treating obesity-induced insulin resistance.

## 1. Introduction

Obesity is a serious global health problem [1, 2]. In the United States, the prevalence of youth and adult obesity is increasing, with an age-adjusted prevalence of 35% in men and 40.4% in women in 2013-2014 [3]. The prevalence of type 2 diabetes mellitus (T2DM) increases along with obesity and has an estimated prevalence of 18.5% in obese adults and 5.4% in normal-weight adults in the United States in 2013-2014 [4]. T2DM is a serious chronic metabolic disease triggered by impaired insulin signal pathways and systemic insulin resistance and the lack of response to insulin target cells such as hepatocytes, skeletal muscle cells, and adipocytes [5]. Insulin resistance occurs during the development of metabolic abnormalities and diseases, including T2DM, hypertension, and dyslipidemia, and decreasing insulin resistance improves metabolic control in T2DM patients [6].

Activated insulin receptor substrate-2 (IRS-2) regulates glucose homeostasis [7]. It transduces insulin action by stimulating the phosphoinositide 3-kinases/protein kinase B (PI3K/AKT) pathway and promotes glucose uptake by insulin-sensitive glucose transporter type 4 (GLUT4) in the plasma membrane. Glucose transport fails because of insulin resistance in T2DM. Defective GLUT4 transport is a feature of insulin resistance, which is a precursor of T2DM [8]. MicroRNAs (miRNAs) are small noncoding RNA molecules that consist of approximately 23 nucleotide pairs [9]. They have been reported to influence adipogenesis and fat metabolism, and differential expression of miRNAs has been reported in tissues from obese versus nonobese people [10]. A significant correlation has been reported between obesity and increased risk of insulin resistance and T2DM [11]. The pathogenesis of insulin resistance is complex and not well understood, but free fatty acids (FFAs) may be involved [12]. An excess of lipids increases circulating FFAs and evokes insulin resistance in muscle and liver tissues [13]. Palmitic acid (PA), a representative FFA, has been shown to directly impair insulin signaling in cultured hepatocytes and myotubes [14]. Palmitic acid (PA) is the most common saturated fatty acid found in animals and plants and is found in foods like meat, cheese, butter, and other dairy products. PA or palmitate at concentrations of 0.4 to 1.0 mM can induce a model of insulin resistance in cultured HepG2 cells [15–17]. Whether miRNAs are involved in the induction of resistance is not yet understood.

In this study, the molecular mechanism of PA-induced insulin resistance was investigated in HepG2 human hepatocyte cells. The aim was to develop novel rationale prevention and treatment of T2DM. We found that PA induced miR-221 expression in HepG2 cells that impaired PI3K/AKT signaling pathway and inhibited glucose uptake.

## 2. Materials and Methods

### 2.1. HepG2 Cell Culture and Glucose Uptake Experiment

HepG2 cells (ATCC, Manassas, VA, USA) were maintained in Dulbecco's modified Eagle medium (DMEM) supplemented with 10% FBS, 100 units/mL streptomycin, and 100 *μ*g/mL penicillin at 37°C in a humidified 5% CO_2_ atmosphere. To assess the glucose uptake, HepG2 cells were transferred to 6-well plates and treated with 0–0.8 mM PA for 24 h. Glucose uptake was assayed by the glucose oxidase method (Bio-Rongsheng, Shanghai, China), following the kit manufacturer's instructions. Glucose oxidase catalyzes the breakdown of glucose to hydrogen peroxide and D-glucono-*δ*-lactone. Peroxidase then catalyzes the formation of a red quinone imide in a reaction involving hydrogen peroxide, 4-aminoantipyrine, and phenol. The absorbance of the resulting solution at a wavelength of 490 nm is proportional to the glucose concentration.

### 2.2. Quantitative Real-Time Polymerase Chain Reaction (qRT-PCR)

HepG2 cells were harvested after treatment and washed three times with ice-cold phosphate-buffered saline. Total RNA was extracted by TRIzol reagent (Invitrogen) following the manufacturer's instructions. The purity and concentration of the extracted RNA were determined at 260/280 nm using a spectrophotometer (NanoDrop ND-1000) and was reverse transcribed into cDNA with a TaKaRa One-Step RT-PCR kit using 1 *μ*g of total RNA and the supplied Oligo (dT) primers. Gene expression was determined by qRT-PCR performed with SYBR Green chemistry and a StepOnePlus system (LightCycler 480 II, Roche). The PCR program started at 95°C for 30 s followed by 40 cycles of denaturation at 95°C for 5 s, annealing at 60°C for 30 s, extension at 95°C for 5 s, and a final extension at 60°C for 1 min. Melting curves were obtained stepwise from 55°C to 95°C. Data were reported as fold change (2^−△△Ct^). Assays were performed independently in triplicate. The primer sequences are shown in Table 1.

### 2.3. miRNA Isolation and Detection

A miRNA cDNA Synthesis Reaction System and a qPCR system (Mir-X™ miRNA First-Strand Synthesis and SYBR® qRT-PCR, TaKaRa Bio Company, USA) were used, following the manufacturer's instructions. The prepared PCR solution, except for the DNA template, was pipetted into 96-well plates. The DNA template of the PCR was added, and the amplification conditions were set. The amplification reaction was performed with a QuantStudio™ 3 and 5 Real-Time PCR System with U6 as a control. The reactions were performed in triplicate. The primer sequences for miRNA-221 and U6 are shown in Table 2.

### 2.4. Luciferase Reporter Assay

Psi-Check2 wild-type 3′-untranslated sequences (wt-3′-UTR) of PI3KR1, containing the miR-221 binding site ligated to a pLuc-reporter luciferase vector, are shown in Supplementary [Supplementary-material supplementary-material-1] and were synthesized by Sangon Biotech (Shanghai, China). PCR was performed with Primer STAR® HS DNA Polymerase (TaKaRa Bio Inc. Shiga, Japan) following the manufacturer's instructions. Psi-Check2 mutated vectors (mut-3′-UTR) were constructed using the Fast Mutagenesis System (TransGen Biotech, China). All constructs were verified by DNA sequencing.

HEK-293T human embryonic kidney cells were seeded in 24-well plates, cotransfected with 10 nmol pre-miR-221 or pre-miR-NC and 100 ng pLuc-3′-UTR. The cells were harvested 24 h after transfection. Luciferase activity was measured with a Dual-Luciferase Reporter Assay System (Promega, USA) and a Glomax Luminometer (Promega, USA). Renilla luciferase activity was normalized to firefly luciferase. All assays were conducted in triplicate.

### 2.5. Cell Transfection

Cells were transfected with Lipofectamine 3000 (Thermo Fisher Scientific, Waltham, MA, USA), following the manufacturer's instructions. The miR-221 mimic and mi-221 inhibitor were purchased from Sangon Biotech (Shanghai, China). The primer sequences are shown in Table 3. PA was added to the cell culture media 24 h after transfection, and cells were cultured for another 24 h.

### 2.6. Western Blotting

Total protein was extracted with RIPA lysis buffer and separated by sodium dodecyl sulfate-polyacrylamide gel electrophoresis (SDS-PAGE). The proteins were transferred to polyvinylidene difluoride membranes, blocked with 5% skim milk in Tris-buffered saline containing 0.1% Tween-20 for 1 h, and then incubated with anti-PI3K, anti-AKT, anti-p-PI3K, anti-p-AKT, and anti-*β*-actin primary antibodies (Proteintech, Rosemont, IL, USA) for 16 h at 4°C. Membranes were washed three times in Tris-buffered saline containing 0.1% Tween-20 and then incubated with anti-mouse or anti-rabbit IgG secondary antibodies for 1 h. Immunoreactive bands were visualized by a commercial electrochemiluminescence (ECL) kit (Amersham Pharmacia Biotech, Little Chalfont, Great Britain).

### 2.7. Statistical Analysis

Values were expressed as the means ± standard deviation (SD). Between-group differences were compared with unpaired two-tailed *t*-tests. Multigroup differences were compared by analysis of variance with Dunnett's multiple comparison test or the Tukey–Kramer test. *p* values <0.05 were considered statistically significant.

## 3. Results

### 3.1. PA Decreased Glucose Uptake in HepG2 Cells

**Figure 1:**
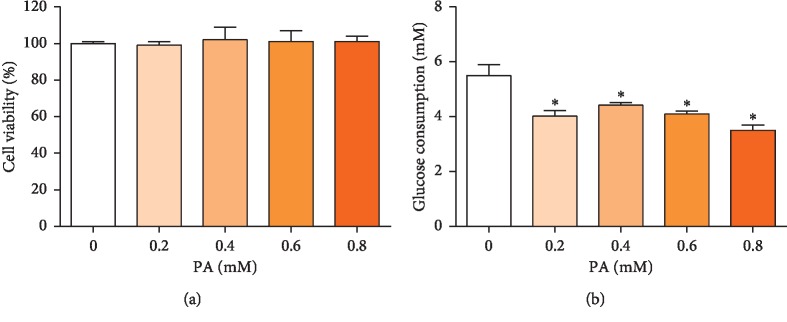
Effects on HepG2 cell viability (a) and glucose uptake (b) after treatment with 0–0.8 mM PA treatment for 24 h. Values are the means ± SD (*n* = 3). ^*∗*^*p* < 0.05, versus 0 mM PA.

PA has been shown to directly impair insulin signaling in cultured hepatocytes and has been previously used to induce insulin resistance in HepG2 cells [15, 16]. As shown in Figure 1, treatment with 0.2 to 0.8 mM PA for 24 h did not inhibit cell viability and the lowest glucose uptake occurred with 0.8 mM PA. Consequently, 0.8 mM PA for 24 h was used to establish the HepG2 insulin resistance model.

### 3.2. PA Impairs PI3K/AKT Signaling Pathway and Increases miR-221 Expression

The PI3K/AKT pathway regulates glucose homeostasis via insulin-sensitive GLUT4 [18]. In this study, inhibition of the PI3K/AKT pathway was determined by assay of IRS-2, PI3K, AKT, and GLUT4 mRNA expression in HepG2 cells, before and after exposure to PA for 24 h. IRS-2 mRNA expression was significantly increased by PA, but PI3K, AKT, and GLUT4 mRNA expressions were decreased. Normally, PI3K is upregulated by IRS-2, but that was not observed in this study, possibly because of the change in miRNA expression [19].

**Figure 2:**
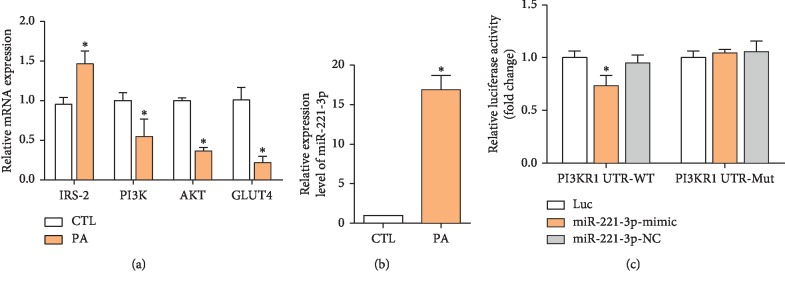
Effect of PA on the expression of PI3K/AKT-related mRNAs and miR-221. IRS-2, PI3K, AKT, and GLUT4 mRNA expressions were assayed in HepG2 cells after treatment with 0.8 mM PA for 24 h (a); PA significantly increased miR-221 expression compared with controls (b); luciferase reporter assay demonstrated PI3K binding of miR-221 by PI3K (c). Values are the means ± SD (*n* = 3). ^*∗*^*p* < 0.05, versus control (a and b) or UTR-Mut (c).

miRNAs regulate gene expression posttranscriptionally by directly binding to the 3′-untranslated region (3′-UTR) of target mRNAs [9]. Dysregulation of miRNAs has previously been implicated in the pathogenesis of various diseases, including obesity and diabetes [20]. miR-221 is involved in the development of obesity and has been reported to negatively regulate insulin sensitivity [21]. qPCR confirmed that PA significantly increased miR-221 expression (Figure 2(b)).

To verify that PI3K was a direct target of miR-221, the putative miR-221-binding sequence at the 3′-UTR of the PI3K gene was subcloned into a luciferase reporter vector. Ectopic expression of miR-221 significantly reduced luciferase activity in HepG2 cells transfected with the reporter vector containing the PI3K 3′-UTR sequence. The effect of miR-221 was abolished when the binding sequence was mutated (Figure 2(c)).

### 3.3. miR-221 Expression Decreases Glucose Uptake in HepG2 Cells

**Figure 3:**
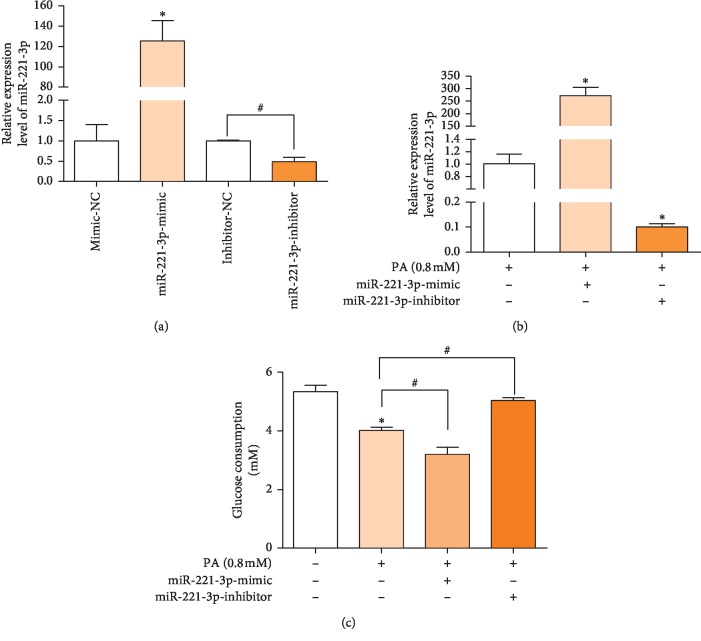
Effect of miR-221 mimic and inhibitor on miR-221 transfection on miRNA expression and glucose uptake. miR-221 expression was assayed by qPCR. (a); HepG cells were transfected with miR-221 mimic or inhibitor for 24 h PA was added to culture media for 24 h and miR-221 expression was assayed (b); glucose consumption was determined by the glucose oxidase method (c). Values are the means ± SD (*n* = 3). ^*∗*^*p* < 0.05, versus control group, ^#^*p* < 0.05, versus the indicated group (*n* = 3).

The involvement of miR-221 in glucose uptake by HepG2 cells treated with PA was investigated in HepG2 cells transfected with miR-221 mimic and inhibitor. The expression of miR-221 was significantly increased in HepG2 cells transfected by mimic and was decreased in those transfected by inhibitor with (Figure 3(a)) and without (Figure 3(b)) PA treatment. As shown in Figure 3(c), miR-221 mimic significantly reduced glucose transport by HepG2 cells and miR-221 inhibitor reversed the reduction in response to PA, indicating that miR-221 modulated glucose uptake.

### 3.4. miR-221 Suppresses GLUT4 Expression via the PI3K/AKT Pathway

**Figure 4:**
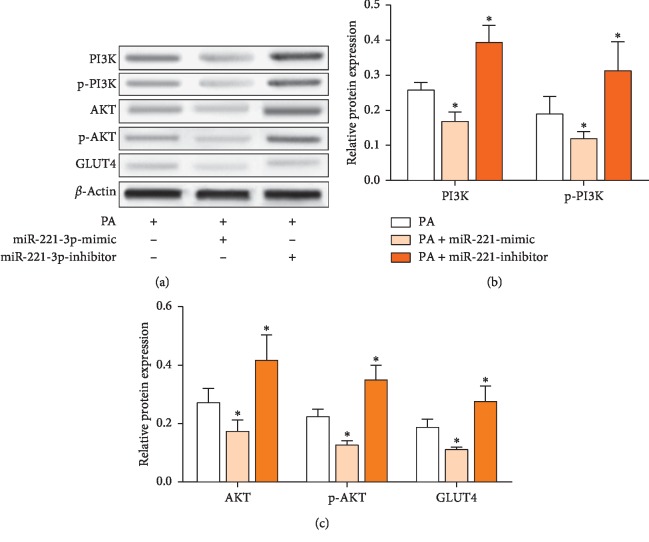
Effect of miR-221 mimic and inhibitor on expression of PI3K/AKT pathway proteins. HepG cells were transfected with miR-221 mimic or inhibitor for 24 h; PA was then added for an additional 24 h. PI3K, p-PI3K, AKT, p-AKT, and GLUT4 protein expressions were assayed by immunoblotting (a), normalized against *β*-actin, and expressed as fold change compared with PA-treated controls (b, c). Values are the means ± SD (*n* = 3). ^*∗*^*p* < 0.05, versus control group.

Changes in protein expression indicated that the effects of miR-221 on glucose transport were mediated by the PI3K/AKT pathway.  Figure 4 shows that PI3K, p-PI3K, AKT, p-AKT, and GLUT4 protein expressions were decreased by miR-221 mimic and that the miR-221 inhibitor blocked the effects of PA on protein expression. The results indicate that miR-221 regulated glucose uptake via the PI3K/AKT pathway.

## 4. Discussion

Overweight and obesity are both increasing in prevalence worldwide. Most patients with T2DM are overweight or obese and have elevated plasma FFA levels that are correlated with the severity of insulin resistance [22, 23]. Consumption of a diet high in saturated versus monounsaturated fats has been associated with increased insulin resistance [24]. High levels of circulating FFAs are thought to have a key role in initiating and promoting the progression of insulin resistance, but the mechanism of action is not fully understood. In this study, PA, a representative FFA, induced the expression of miR-221, which then bound to PI3K mRNA to inhibit its expression, and that of PI3K/AKT pathway-associated proteins to decrease glucose uptake.

IRS-2 is a cytoplasmic adaptor protein that organizes signaling complexes downstream of cell surface receptors and it coordinates responses to insulin that are associated with induction and worsening of resistance [25]. PI3K is an intracellular signal transducer of diverse cellular functions. AKT is a protein kinase downstream of PI3K that mediates the release of growth factors, cytokines, and other stimuli of cell survival, growth, proliferation, angiogenesis, and metabolism. The IRS/PI3K/AKT signaling pathway regulates insulin signaling and lipid metabolism [19, 26]. In this study, PA induced the expression of IRS-2 but not PI3K, its downstream target. The lack of PI3K response was mediated by miR-221.

There is evidence that miRNAs are involved in insulin secretion, *β*-cell differentiation, and glycolipid metabolism and that they contribute to the progression of T2DM [27, 28]. Previous studies have described the involvement of miR-221 in the development of obesity and decreased insulin sensitivity [21, 29]. In the previous study, postnatal overfeeding was found to induce miR-221 expression and impaired PI3K/AKT signaling in the livers of adult male rats. In the present study, we found that miR-221 influenced IRS/PI3K/AKT signaling during the induction of insulin resistance by PA.

miR-221 is located on the X chromosome (Xp11.3). It is highly conserved in vertebrates and is a putative oncogene that is overexpressed in a number of human tumors [30–32]. Adipose miR-221 is upregulated in obesity, and its expression is positively correlated with an increased body mass index in the Pima Indian population, which has a high prevalence of T2DM [33]. The luciferase reporter assay revealed that in HepG2 cells transfected by miR-221 mimic and inhibitor, PA induced the expression of miR-221 by binding to PI3K mRNA and suppressing its expression. Thus, miR-221 played an important role in the insulin resistance induced by PA.

GLUT4 is an insulin-regulated transmembrane glucose transporter that controls glucose homeostasis and is a downstream target of the PI3K/AKT pathway [18]. In this study, PA decreased glucose uptake by suppressing GLUT4 expression. The decreased GLUT4 expression can be accounted for by the impairment of the PI3K/AKT signaling pathway. Polysaccharides extracted from *Enteromorpha prolifera*, an edible seaweed, have hypoglycemic and hypolipidemic effects and have been shown to lower blood glucose by increasing GLUT4 expression [34–38]. Thus, polysaccharides from dietary seaweed may be found effective for preventing T2DM.

**Figure 5:**
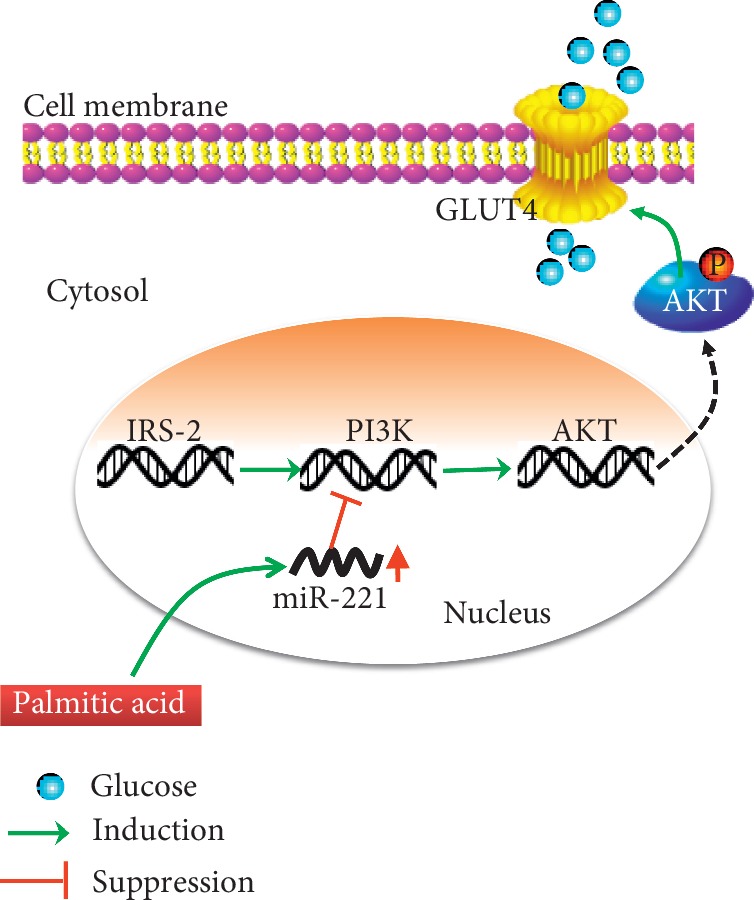
Proposed mechanism of PA induction of insulin resistance in HepG2 cells. PA induces miR-221 overexpression. miR-221 binds PI3K to decrease PI3K and AKT expression and phosphorylation. Impairment of the PI3K/AKT pathway results in reduced GLUT4 expression and glucose consumption, which induces insulin resistance.

In summary, PA decreased glucose uptake in HepG2 cells by promoting miR-221 overexpression, which reduced GLUT4 expression and glucose uptake by impairing PI3K/AKT signaling. A proposed mechanism of miR-221 overexpression induced by PA and the resulting impairment of the PI3K/AKT pathway is shown in Figure 5. The study results suggest that inhibition of miR-221 may have potential as a novel approach for preventing and treating obesity-induced insulin resistance.

## Figures and Tables

**Table 1:** The primer sequences of IRS-2, PI3K, and GLUT4.

Gene	Primer sequence	
	Forward	Reverse
IRS-2	CACCTACGCCAGCATTGACTTC	CAAACACAGTCATTGCTCAGATCC
PI3K	AGCATTGGGACCTCACATTACACA	ACTGGAAACACAGTCCATGCACATA
AKT	AGCGACGTGGCTATTGTGAA	CACGTTGGTCCACATCCTG
GLUT4	GGGCTGAGACAGGGACCATAAC	CATGAGCAATGGCATCCCAGAA
*β*-Actin	TGGATCAGCAAGCAGGAGTA	ATGGTGGTGAAGACGCCAGTA

**Table 2:** The primer sequences of U6 and miR-221.

Name of miRNA		Sequence of primer
Hsa-miR-221-3p	Forward	CTACATTGTCTGCTGGGTTTC
U6	Forward	GGAACGATACAGAGAAGATTAGC
	Reverse	TGGAACGCTTCACGAATTTGCG

**Table 3:** The sequences of miR-221-3p-mimic/inhibitor.

Name of miRNA	Sequence of primer
Hsa-miR-221-3p-mimic	AGCUACAUUGUCUGCUGGGUUUC
Has-miR-221-3p-inhibitor	GAAACCCAGCAGACAAUGUAGCU

## Data Availability

The data used to support the findings of this study are available from the corresponding author upon request.

## Abbreviations

IRS-2: Insulin receptor substrate-2
PI3K: Phosphoinositide 3-kinase
AKT: Protein kinase B
GLUT4: Glucose transporter type 4
MicroRNA: miRNA
T2DM: Type 2 diabetes mellitus.

## Supplementary Materials

Supplementary Materials Supplementary Figure 1: a schematic diagram showing the construction of the luciferase reporter vectors.
