# Supplementary material for: Palmitic Acid Induces MicroRNA-221 Expression to Decrease Glucose Uptake in HepG2 Cells via the PI3K/AKT/GLUT4 Pathway
Source: Biomed Res Int. 2019 Nov 11;2019:8171989. doi: 10.1155/2019/8171989 (PMC6885153; doi:10.1155/2019/8171989)
Supplement: Supplementary Materials — Supplementary Figure 1: a schematic diagram showing the construction of the luciferase reporter vectors. [file 8171989.f1.pdf]

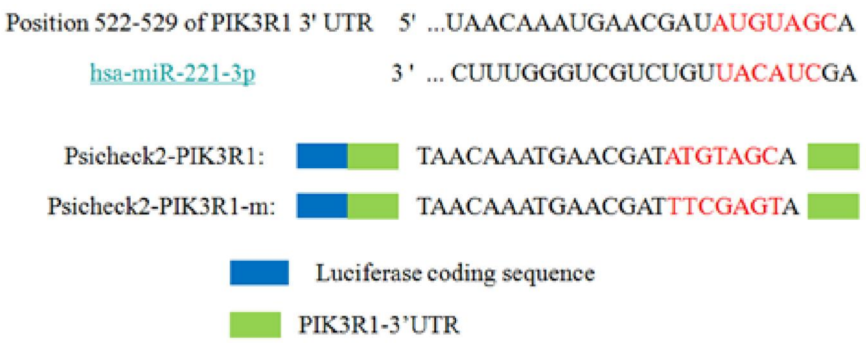

Supplemental Figure 1. A schematic diagram showing the construction of the luciferase reporter vectors
